# Flash Glucose Monitoring for Predicting Cardiogenic Shock Occurrence in Critically Ill Patients: A Retrospective Pilot Study

**DOI:** 10.3390/diagnostics15060685

**Published:** 2025-03-11

**Authors:** Velimir Altabas, Dorijan Babić, Anja Grulović, Tomislav Bulum, Zdravko Babić

**Affiliations:** 1 Department of Endocrinology, Diabetes and Metabolic Diseases, Sestre Milosrdnice University Clinical Hospital, 10000 Zagreb, Croatia; 2 School of Medicine, University of Zagreb, 10000 Zagreb, Croatia; dorijanbabic@gmail.com (D.B.); zbabic@net.hr (Z.B.); 3 Department of Diabetes and Endocrinology, Vuk Vrhovac University Clinic for Diabetes, Endocrinology and Metabolic Diseases, Merkur University Hospital, 10000 Zagreb, Croatia; 4 Coronary Care Unit, Department of Cardiology, Sestre Milosrdnice University Clinical Hospital, 10000 Zagreb, Croatia

**Keywords:** hypoglycaemia, cardiogenic shock, flash glucose monitoring, critical care

## Abstract

**Background/Objectives:** Continuous and flash glucose monitoring (CGM and FGM) may enhance glucose management by providing real-time glucose data. Furthermore, growing evidence is linking altered blood glucose concentrations and worse short-term outcomes in critically ill patients. While hyperglycemia is more common in these patients and is associated with an increased risk of adverse events, hypoglycemia is particularly concerning and significantly raises the risk of fatal outcomes. This exploratory study investigated the link between FGM variables and cardiogenic shock in critically ill Coronary Care Unit (CCU) patients. **Methods:** Twenty-eight CCU patients (1 May 2021–31 January 2022) were monitored using a Libre FreeStyle system. Analyzed data included patient demographic and laboratory data, left ventricular ejection fraction, standard glucose monitoring, APACHE IV scores, and cardiogenic shock occurrence. Analysis was performed using the χ^2^ test, Mann–Whitney U test, and logistic regression. **Results:** Among the patients, 13 (46.43%) developed cardiogenic shock. FGM detected hypoglycemia in 18 (64.29%) patients, while standard methods in 6 (21.43%) patients. FGM-detected hypoglycemia was more frequent in patients who developed cardiogenic shock (*p* = 0.0129, χ^2^ test) with a significantly higher time below range reading (*p* = 0.0093, Mann Withney U test), despite no differences in mean glucose values. In addition, hypoglycemia detected by FGM was an independent predictor of shock (*p* = 0.0390, logistic regression). **Conclusions:** FGM identified more hypoglycemic events compared to standard glucose monitoring in the CCU. Frequent FGM-detected hypoglycemic events were associated with cardiogenic shock, regardless of a history of diabetes. Due to a limited sample size, these results should be interpreted cautiously and further research in this area is justified.

## 1. Introduction

Continuous glucose monitoring (CGM) and flash glucose monitoring (FGM) have revolutionized diabetes management by enabling better monitoring of glucose levels, providing deeper insight into glycemic fluctuations, and capturing more hypoglycemic and hyperglycemic events compared to standard blood glucose monitoring. These systems measure glucose levels in the subcutaneous interstitial fluid rather than in capillary blood, which is traditionally assessed through the self-monitoring of blood glucose using glucometers and fingersticks. Glucose readings from CGM and FGM are typically updated every 1–5 min, generating a significant volume of data. Beyond its enhanced sensitivity in detecting clinically relevant glucose level excursions, this wealth of information has facilitated the development of advanced metrics such as time in range (TIR), time in tight range (TITR), time above range (TAR), time below range (TBR), and glucose variability (GV) [1,2]. These derived parameters correlate with the long-term occurrence of chronic diabetic complications and associated vascular diseases, regardless of the type of diabetes [3,4,5].

In inpatient settings, glucose control has its specificities, since several other factors need to be considered, such as concomitant disease and medication, including adjustments in antidiabetic drugs, limited food intake, and decreased physical activity. Over time, glucose monitoring systems have been recognized as a valuable tool for monitoring glucose control and adjusting antidiabetic treatment in hospitalized patients [6,7]. However, there is still no widely accepted consensus regarding treatment targets for this sort of monitoring in hospitalized patients [8,9]. This lack of consensus may hinder the treatment of this patient group. Especially in critically ill patients, blood glucose fluctuations and their association with adverse outcomes necessitate close monitoring and intervention. There is mounting evidence of a consistent association between altered blood glucose concentration parameters detected by glucose monitoring systems and worse short-term outcomes in critically ill patients [10,11]. These findings align with the existing knowledge about the harmful impact of both hyperglycemia and hypoglycemia on the outcomes in various diseases such as acute coronary syndrome, stroke, pulmonary embolism, sepsis, and injuries. In these acute conditions, blood glucose levels may be elevated even in patients with otherwise normal glucose metabolism due to acute hormonal and inflammatory dysregulations, a phenomenon known as stress hyperglycemia. These patients may also experience hypoglycemia [12,13,14,15].

Several mechanisms contribute to increased glucose fluctuations and, consequently, to variations in energy supply. In critically ill patients, counterregulatory hormones such as catecholamines, cortisol, glucagon, and growth hormone are elevated, disrupting glucose homeostasis. Moreover, a simultaneous rise in inflammatory cytokines further worsens the metabolic environment [12,16]. It is important to emphasize that while hyperglycemia is more common in these patients and is associated with an increased risk of adverse events, hypoglycemia is particularly concerning and significantly raises the risk of fatal outcomes more than hyperglycemia does [17]. Therefore, slightly higher blood glucose targets for traditional blood glucose monitoring (between 7 and 10 mmol/L) are recommended for diabetic patients in inpatient settings compared to outpatient settings to minimize the risk of hypoglycemia, except for selected patients at a low risk of hypoglycemia [18].

## 2. Cardiogenic Shock and Hypoglycemia

Among other conditions, cardiogenic shock is a serious one associated with severe hormonal derangements and elevated inflammatory cytokines. It is a severe and life-threatening condition characterized by inadequate tissue perfusion resulting from a significant decrease in cardiac output [19,20]. Ventricular failure following acute myocardial infarction (AMI) remains the most common cause of cardiogenic shock. Other causes include heart failure, valvular heart disease, acute myocarditis, Takotsubo syndrome, and arrhythmias [21]. Cardiogenic shock is primarily triggered by the inability to maintain adequate stroke volume despite sufficient preload, leading to reduced cardiac output and tissue hypoperfusion. Early compensatory mechanisms, such as peripheral vasoconstriction and fluid retention, initially aim to preserve organ perfusion but worsen the preload and afterload mismatch, exacerbating congestion and diastolic dysfunction. Key pathophysiological features include decreased cardiac index, elevated pulmonary capillary wedge pressure, increased central venous pressure, and neurohormonal activation (e.g., catecholamine release and renin–angiotensin–aldosterone system activation). Adrenergic vasoconstriction increases afterload and wall stress, further reducing cardiac output. Hypoxic tissue damage triggers the release of inflammatory cytokines, compounded by factors like microbial translocation or ischemia–reperfusion injury. This causes microcirculatory dysfunction, resulting in either “warm and wet” or mixed shock phenotypes. Furthermore, pulmonary congestion and edema impair oxygen and carbon dioxide exchange, increase right ventricular afterload, and worsen myocardial ischemia, further reducing ventricular function. If interventions fail to restore cardiac output, progressive hypoperfusion leads to multi-organ failure, often dominated by metabolic derangements such as acute kidney injury and congestive liver damage [22,23]. Notably, some of these mechanisms can influence blood glucose control [20,24].

In addition, altered cardiac energy metabolism and demand contribute to decreased ventricular function in cardiogenic shock. In the healthy heart, various energy sources are utilized, including carbohydrates (glucose and lactate), fatty acids, ketones, and amino acids. In healthy hearts, fatty acid oxidation provides approximately 40–60% of ATP production, carbohydrate metabolism (glucose and lactate) contributes 20–40%, ketone oxidation accounts for 10–15%, and amino acid oxidation makes up less than 2% [25]. The failing heart shifts from fatty acid metabolism to glucose metabolism, particularly relying on glycolysis for adenosine triphosphate (ATP) production. This finding is supported by human metabolomic studies using heart samples from patients with heart failure with reduced ejection fraction (HFrEF) who received a left ventricular assist device. These studies demonstrated increased glucose uptake and upregulated glycolytic rates in myocardial samples from HFrEF patients compared to non-failing controls [25,26]. Thus, glucose availability and appropriate blood glucose concentrations appear to be critically important during acute cardiac conditions, including cardiogenic shock. Severe hypoglycemia with the subsequent cardiogenic shock has been documented in case reports, although the exact pathogenesis is still unclear [27].

However, hypoglycemia is less frequently detected than hyperglycemia with traditional inpatient blood glucose monitoring and is commonly a result of hyperglycemia overtreatment, occurring in less than 3% of hospitalized patients. Spontaneous hypoglycemia has been reported in patients with multiple organ failure, though its exact incidence remains unknown. Risk factors include, but are not limited to, the patient’s age, severe comorbid diseases like anemia, liver failure, heart failure, and other endocrine disorders [28]. As mentioned earlier, hypoglycemic events are linked to a poor prognosis [17,29].

This exploratory study aimed to investigate the correlation between hypoglycemia detected by FGM and the occurrence of cardiogenic shock in a cohort of critically ill patients admitted to the Coronary Care Unit (CCU).

## 3. Materials and Methods

### 3.1. Study Subjects

A cohort of 28 critically ill patients admitted to the CCU from 1 May 2021 to 31 January 2022 was enrolled in this study. The inclusion criterion was a critical heart condition without shock as the reason for hospitalization in the CCU. Therefore, patients who were admitted primarily for cardiogenic shock and those with a stay in the CCU shorter than 48 h were excluded from the study. Additionally, patients younger than 18 years and pregnant women were also excluded. Patients were considered to have diabetes based on their medical history or HbA1c levels at the time of hospitalization. Informed consent was obtained from/for all subjects involved in the study. The study was approved by the local ethics committee and conducted according to the Declaration of Helsinki.

### 3.2. Glycemic and Vital Signs Measurements

At CCU admission, clinical status and laboratory findings were assessed, and the Acute Physiology and Chronic Health Evaluation (APACHE) IV score was calculated [25]. Left ventricular ejection fraction (LVEF) was determined by ultrasound using the Simpson method. All patients were treated for their medical conditions according to contemporary guidelines. Standard 7-point glucose profiles with traditional fingerstick methods were conducted for each patient throughout the hospitalization. At the same time, each patient received a Libre FreeStyle sensor (Abbott Laboratiories, Lake County, IL, USA) for flash glucose monitoring. Cut-offs for normal tissue glucose values were set at 3.9–10.0 mmol/L. A trained nurse regularly scanned the patients during the entire hospitalization, capturing virtually all glucose readings. FGM was discontinued either upon hospital discharge or in the event of the patient’s death.

Glucose concentration validation was performed regularly, 7 times a day, and additionally upon detecting a hypoglycemic event. FGM-detected hypoglycemic events had to be verified using either fingerstick capillary glucose measurements or standard laboratory full blood glucose readings. In case of discrepancies (e.g., a false positive hypoglycemia reading on FGM), the standard blood glucose measurement was used to categorize the event. Vital signs and parameters, as well as clinical evolution, were monitored, with cardiogenic shock occurrence (stage C or worse according to the SCAI clinical expert consensus) as a prespecified outcome [30].

### 3.3. Statistical Analysis

Descriptive statistics were performed using percentages for qualitative data, and medians and interquartile ranges for quantitative data. Data were further analyzed by using the χ^2^ or Mann–Whitney U test for univariate analysis, and logistic regression for multivariate analysis. Data analysis was conducted using Medcalc 22.019.

## 4. Results

The age of the enrolled patients ranged from 62 to 90 years (median age 75 years). Out of the 28 patients included in this study 20 were men (71.43%).

The patients were admitted to the CCU due to acute myocardial infarction (thirteen patients; 46.43%), acute heart failure (five patients; 17.86%), out-of-hospital cardiac arrest (two patients; 7.14%), endocarditis and/or pericarditis (six patients; 21.43%), and valvular disease (two patients; 7.14%).

Eighteen patients had type 2 diabetes mellitus (64.29%). Among them, the majority were on oral antidiabetics (ten patients, 55.56%), while the others received insulin in combination with oral therapy (eight patients, 44.44%), as part of their home treatment. Previously diagnosed liver cirrhosis was present in a single patient, while previously known chronic kidney disease, according to KDIGO guidelines, was present in 19 patients [31].

The duration of FGM ranged from 3 to 38 days for the entire study group. Specifically, it ranged from 3 to 25 days in the group without FGM-detected hypoglycemia and from 3 to 38 days in the group with FGM-detected hypoglycemia.

The relevant baseline patient data with the results of the univariate analysis are shown in Table 1.

When the study cohort was divided into groups based on the presence of hypoglycemia readings detected by FGM, there was no significant difference between the groups regarding hypoglycemia detection using standard fingerstick glucose profiles (*p* = 0.4887, χ^2^ test). However, FGM recognized hypoglycemia in more patients than standard glucose monitoring (18 vs. 5 patients). Notably, in all patients with FGM-recognized hypoglycemia, confirmatory low blood glucose concentrations were detected at least once. In one patient, hypoglycemia was detected by standard methods but was not recorded by flash glucose monitoring. This patient had favorable clinical outcomes, with no occurrence of shock or a fatal outcome.

There was no significant difference between groups in age, APACHE IV score, length of hospitalization, history of hypertension, diabetes, in-hospital insulin use, coronary artery disease, congestive heart failure, chronic renal insufficiency, and liver disease. At admission, there was no significant difference regarding systolic blood pressure, pulse, admission blood glucose, hemoglobin values, serum creatinine, or left ventricular ejection fraction. Notably, females had significantly fewer hypoglycemic events detected by FGM (*p* = 0.0144).

In the study, in the group experiencing hypoglycemia recognized by FGM, mean blood glucose levels were significantly lower than in the group without hypoglycemia (*p* = 0.0214). Additionally, the TBR was significantly higher in the group with hypoglycemia (*p* < 0.0001). The group with FGM-recorded hypoglycemia developed cardiogenic shock more frequently and this difference was significant (*p* = 0.027, χ^2^ test). However, fatal outcomes did not occur significantly more frequently in either group.

When the study cohort was grouped based on cardiogenic shock occurrence for further analysis, a significant correlation was found between shock occurrence and left ventricular ejection fraction (*p* = 0.016) as well as hypoglycemia recognized by FGM (*p* = 0.013). Additionally, a higher TBR reading was significantly associated with shock development (*p* = 0.009). In addition, study participants who developed shock were significantly more likely to die (*p* = 0.043). There was no significant correlation between the other analyzed parameters and the occurrence of shock.

The results including the univariate analysis are shown in Table 2.

When logistic regression was used for the multivariate analysis, both the hypoglycemia readings detected by FGM and the left ventricular ejection fraction independently contributed to shock occurrence (Table 3). However, when gender was included in the analysis, it did not show an independent contribution to shock occurrence (Table 4).

## 5. Discussion

Hypoglycemia is a common occurrence in patients with various forms of diabetes and is primarily caused by an imbalance between glucose supply and antidiabetic treatments, particularly insulin, sulfonylureas, and glinides. Notably, insulin is widely prescribed in inpatient settings for diabetes treatment. Additional contributing factors may include increased glucose consumption, altered glucose supply and insulin degradation in hepatic or kidney failure, and, theoretically, heightened insulin sensitivity. However, the role of increased insulin sensitivity in hypoglycemia is less likely in acute states, as these are typically characterized by elevated levels of counterregulatory hormones and proinflammatory cytokines [32,33].

Less is understood about the pathophysiology of hypoglycemia in individuals without diabetes, particularly in life-threatening conditions. Contributing factors may include downregulated gluconeogenesis due to elevated cytokine levels, liver and kidney dysfunction, depletion or damage of glucose stores, a blunted counterregulatory hormone response, and increased glucose consumption during hypoxia. Hypoglycemia activates sympathoadrenal mechanisms, disrupts cardiac repolarization, dysregulates hemostatic processes—including accelerated thrombogenesis and vasoconstriction—and further elevates inflammatory cytokine levels, ultimately worsening patient outcomes [33,34]. Despite the observed association between hypoglycemia and increased mortality, some landmark studies could not prove a direct causal relationship between hypoglycemia and cardiovascular events. For instance, in the ADVANCE trial, no clear correlation was found between repeated episodes of severe hypoglycemia and adverse cardiovascular outcomes. This suggested that hypoglycemia may not have directly caused cardiovascular events but could instead serve as a marker of underlying vulnerability in critically ill patients [35]. In the ACCORD trial, intensive glucose control improved glucose metabolism parameters but led to more frequent hypoglycemia, which was associated with increased mortality [36]. Paradoxically, in the intensively treated group in the ACCORD study, a small but significant inverse relationship was observed between the number of hypoglycemic episodes and the risk of death, although its clinical significance remains uncertain [37]. However, it is important to note that continuous glucose monitoring systems were not available during the time of the study, and a large number of hypoglycemic events may have been unrecognized [38]. Notably, the FGM system is able to detect more hypoglycemic events than standard glucose monitoring and some CGM systems [39,40].

Nevertheless, in experimental models, elevated catecholamines due to hypoglycemia have been associated with blood coagulation abnormalities, including the activation of platelets and coagulation factor VII. This prothrombotic state is further compounded by a proinflammatory response, characterized by increased levels of C-reactive protein (CRP), vascular endothelial growth factor (VEGF), and interleukin-6 (IL-6). Together, these changes may contribute to heightened cardiovascular risk and systemic inflammation, potentially linking hypoglycemia to adverse outcomes in vulnerable patients [41]. Further supporting the notion of hypoglycemia’s adverse effects on the cardiovascular system, Rana et al. demonstrated that hypoglycemia significantly reduced the myocardial blood flow reserve in both healthy individuals and subjects with type 1 diabetes during hypoglycemic clamp studies. This suggests that hypoglycemia may impair coronary perfusion, potentially contributing to cardiovascular complications [42]. At the cellular level, hypoglycemia’s impact on endothelial function has been observed in human umbilical vein endothelial cells (HUVECs). Under hypoglycemic conditions, HUVECs exhibit decreased production of nitric oxide—a critical molecule for vascular relaxation and health—while simultaneously increasing levels of superoxide, a reactive oxygen species that promotes oxidative stress and endothelial dysfunction. This further implicates hypoglycemia as a contributor to vascular damage and cardiovascular risk [43].

The mentioned findings highlight the multifaceted impact of hypoglycemia, affecting both macrovascular and microvascular function, and underscore the importance of avoiding hypoglycemic episodes, especially in vulnerable populations. The results of the present study come along with previous reports that critically ill patients are prone to developing hypoglycemia, regardless of their previous diabetes history [38]. This effect becomes more apparent when hypoglycemia is detected through FGM, as this method captures virtually all fluctuations in glucose levels [44].

The relationship between hypoglycemia and adverse outcomes in critically ill patients has been highlighted in several more studies. Research by Krinsley et al. demonstrated that intensive care unit (ICU) patients who experienced hypoglycemia had a higher hospital mortality rate [39]. This was supported by a subsequent Australian study involving nearly 5000 ICU patients, which revealed that even mild to moderate hypoglycemia significantly raised the risk of mortality [45]. Furthermore, the NICE-SUGAR trial found that strict glucose control in intensive care settings resulted in a greater number of hypoglycemic events, which corresponded to higher mortality rates among patients [46]. Overall, these findings suggest that while controlling blood glucose levels is critical in intensive care, there is a delicate balance to be maintained, as hypoglycemia can pose significant risks to patient survival. Nevertheless, hypoglycemic episodes, regardless of their origin, are linked to unfavorable patient outcomes [47,48,49].

Our pilot study found that FGM identifies more hypoglycemic events than traditional blood glucose monitoring in patients who later developed cardiogenic shock. Although this finding has previously been demonstrated in other patient populations, this is the first study to detect it in this specific patient population, with cardiogenic shock as a prespecified outcome [44,50,51]. Notably, inhospital insulin use was not associated with the occurrence of hypoglycemia or cardiogenic shock. This likely reflects the fact that insulin was used to treat hyperglycemia and was carefully dosed to avoid hypoglycemic events, suggesting appropriate insulin dosing practices [52,53]. Therefore, altered gluconeogenesis and increased glucose consumption may play a prominent role in the pathogenesis of hypoglycemia in this group of critically ill patients [32,33].

Otherwise, female gender was associated with less hypoglycemia. This gender dimorphism can be attributed to metabolic differences between males and females, such as different body composition and fat tissue percentage, increased insulin resistance, poorer previous glucose control, and differences in outpatient antidiabetic therapy [54].

Furthermore, our preliminary results suggested an association between hypoglycemia in FGM readings and the occurrence of cardiac shock, independent of other factors, including LVEF, and gender as a pioneering finding in the literature. While LVEF remained a significant contributing factor after analyzing with logistic regression, gender differences showed no significance, likely because women with serious heart conditions are treated less likely to receive advanced therapies such as mechanical circulatory support [55].

Regarding hypoglycemia, it should be noted, that although the results were significant, the wide confidence intervals for hypoglycemia readings in FGM prevent definite statements about effect estimates. Additionally, we were unable to show significant correlations for fatal outcomes between the groups with and without hypoglycemia, likely due to the small sample size and the possibility that critically ill patients from the control group may have died from causes unrelated to cardiogenic shock. It remains unclear whether hypoglycemia is part of the pathophysiological mechanism in cardiogenic shock or merely a marker of metabolic frailty [56,57]. Answering this question could lead to better tailored interventions to improve patient outcomes. Although this was beyond the scope of our study, several assumptions can be made. First, interventional studies are needed, with prompt interventions to raise blood glucose if levels tend to fall below a certain threshold at different stages of shock development [58]. Outcomes like shock deterioration and mortality should be analyzed. This would necessitate a more sophisticated study design, incorporating different glucose cut-offs to prevent hypoglycemia and enhance patient safety. Additionally, since hypoglycemia may result from improper insulin dosing, categorizing them into insulin-induced and non-insulin-induced could be beneficial. Furthermore, hypoglycemia may also stem from the body’s inability to synthesize glucose from lactate due to circulatory collapse, which affects heart and vascular function. In this case, hypoglycemia serves merely as a marker of multi-organ failure, and the focus should be on implementing shock prevention measures. Finally, a combination of these mechanisms may contribute to the pathophysiology and outcomes of cardiogenic shock [55,56,57].

It should be emphasized that cardiogenic shock is a severe, life-threatening condition with a poor prognosis, even with the use of advanced and specialized treatment options typically available in intensive care units. Importantly, shock may develop as the patient’s condition deteriorates. The prevalence of cardiogenic shock in intensive care and coronary care units is approximately 15%, varying slightly depending on the research. Inhospital mortality rates can reach up to 60%, with roughly half of the patients dying within the first 24 h. One-year mortality rates are approximately 50–60%, with the majority of fatal outcomes occurring within the first two months after presentation [59]. Thus, novel tools to guide targeted treatment in the early phase of ICU or CCU admission are needed to improve outcomes, such as machine learning for hypoglycemia prediction and close loop insulin delivery systems [60,61]. Therefore, given the exploratory nature of this analysis, our findings should undergo further validation, and interventional studies are needed.

## 6. Strengths and Limitations

This pilot study has several strengths and limitations that merit consideration.

Most importantly, to the best of our knowledge, this is the first publication to indicate that hypoglycemia in cardiogenic shock is largely unrecognized by standard glucose monitoring. Furthermore, hypoglycemic events detected by FGM could serve as an independent predictor of cardiogenic shock occurrence, which may be associated with poor clinical outcomes and an increased risk of death. So far, enhanced glucose monitoring has provided novel insights into glycemic fluctuations in cardiogenic shock.

However, additional research is needed to further explore glucose metabolism in cardiogenic shock and shock-related glucovariability, as this study has certain limitations. The limitations include its exploratory and non-interventional nature, a small sample size, a heterogeneous sample, a retrospective single center design, and some technical and statistical considerations. The small sample size and limited monitoring time due to short patient survival prevented us from establishing associations between other FGM parameters—such as glucovariability, mean blood glucose, and glucose management indicator (GMI)—and clinical outcomes. For instance, calculating the GMI required a minimum follow-up period of 5 full days. More than a quarter of patients were either discharged or died before this period earlier, rendering the calculation of GMI impossible. For other parameters, such as glucose variability and mean blood glucose, a larger sample size is required to achieve statistical significance and ensure sufficient statistical power. Furthermore, implementing FGM in the critical care setting of cardiogenic shock requires addressing challenges like accurate glucose monitoring during hemodynamic instability and fluid shifts. Therefore, data interpretation and potential interferences from medications, such as vasoactive substances and crystalloid infusions, as well as interventions, need careful consideration. Lastly, the width of confidence intervals suggests caution in interpreting the results and strongly emphasizes the need for future research using externally validated techniques, including multicenter studies, larger patient cohorts and external validation.

## 7. Conclusions

FGM systems in the CCU setting might be beneficial, regardless of the patients’ previous diabetes history, by providing more insight into glucose fluctuations in critically ill patients. Hypoglycemic episodes, although of utmost clinical importance due to their association with unfavorable outcomes, are relatively seldom recognized and studied. New glucose monitoring technologies, including FGM and CGM, may provide deeper insight into glycemic fluctuations in critically ill patients. These technologies can help improve glucose variability and reduce the number and severity of hyperglycemic and hypoglycemic episodes, potentially having a positive effect on patient survival. Additionally, these systems can enhance outcomes beyond glycemic control by reducing nursing workload, improving staff satisfaction, optimizing healthcare workflows, minimizing patient discomfort, and ultimately increasing patient satisfaction [62]. To summarize, in this small, retrospective pilot study, poor glucose control, indicated by frequent hypoglycemia occurrence and inadequate TBR readings, correlated with patients’ clinical deterioration and progression to cardiogenic shock. Due to previously mentioned limitations and the lack of interventional larger studies, these preliminary results should be interpreted with caution and further investigation is warranted. For now, FGM has the potential to improve blood glucose control and enable more precise and individualized care in the critical care setting.

## Figures and Tables

**Table 1:** Baseline data of the study population.

	With Hypoglycemia Registered with FGM (*N* = 18)	Without Hypoglycemia Registered with FGM (*N* = 10)	Total Population	*p* (Mann Withney U Test, * χ^2^ Test)
Age (years)	75 (IQR 71–81)	75 (IQR 67–81)	75 (IQR 70–79)	0.9283
Female gender	2 (11.11%)	6 (60%)	8 (28.57%)	0.0144 *
Length of hospitalization (days)	17 (IQR 7–24)	9 (IQR 6–13)	9 (IQR 6–21)	0.2585
History of diabetes	9 (50.00%)	9 (90.00%)	18 (64.29%)	0.0917 *
History of hypertension	11 (61.11%)	7 (70.00%)	18 (64.29%)	0.7961 *
Coronary artery disease	13 (72.22%)	5 (50.00%)	18 (64.29%)	0.2044 *
Chronic heart failure	13 (72.22%)	6 (60.00%)	19 (67.86%)	0.6383 *
Chronic renal disease	12 (66.67%)	7 (70.00%)	19 (67.86%)	0.8367 *
Liver disease	0 (0%)	1 (10.00%)	1 (3.57%)	0.6961 *
Systolic blood pressure at admission (mmHg)	130 (IQR 110–150)	120 (IQR 110–145)	125 (IQR 110–150)	0.9045
Pulse at admission (min^−1^)	90 (IQR 80–105)	85 (IQR 70–107)	90 (IQR 76–105)	0.5619
Hemoglobin at admission (g/L)	128 (IQR 99–136)	110 (IQR 91–140)	121 (IQR 99–140)	0.5485
Blood glucose at admission (mmol/L)	12.5 (IQR 8.0–13.1)	10.1 (IQR 7.2–14.4)	11.5 (IQR 7.2–13.1)	0.2585
Serum creatinine at admission (μg/L)	105 (IQR 94–134)	149 (IQR 109–226)	125 (IQR 95–186)	0.1074
APACHE IV score	63 (IQR 54–72)	57 (IQR 45–72)	62 (IQR 49–80)	0.7414
Ejection fraction (%)	35 (IQR 30–50)	42 (IQR 35–50)	40 (IQR 30–50)	0.2041
Hypoglycemia in standard glucose monitoring	5 (27.78%)	1 (10.00%)	6 (21.43%)	0.4887 *
Mean FGM glucose (mmol/L)	6.5 (IQR 5.1–7.7)	7.8 (IQR 7.4–9.1)	7.2 (IQR 5.4–9.2)	0.0214
glucovariability	23.7 (IQR 20.5–33.6)	21.9 (IQR 16.8–28.3)	23.7 (IQR 18.5–29.3)	0.4413
TIR/%	78 (IQR 53–94)	65 (IQR 27–97)	78 (IQR 51–94)	0.9442
TBR/%	8 (IQR 3–19)	0 (IQR 0–0)	2 (IQR 0–11)	<0.0001
TITR/%	68 (IQR 36–79)	57 (IQR 15–68)	65 (IQR 20–79)	0.3030
In-hospital insulin use	11(61.11%)	7(70.00%)	18(64.29%)	0.6381 *
Shock occurence	12 (66.67%)	1(10.00%)	13 (46.43%)	0.0268 *
Death	10 (55.56%)	7 (70.00%)	17 (60.71%)	0.1198 *

Legend: APACHE IV: Acute Physiology and Chronic Health Evaluation (APACHE) IV; TIR: Time in range; TBR: Time below range; TITR: Time in tight range; * χ^2^ Test.

**Table 2:** Correlation of shock occurrence with clinical and laboratory parameters.

	Patients Who Developed Shock (*N* = 13)	Patients Who Did Not Develop Shock (*N* = 15)	Total Population (*n* = 28)	*p* (Mann Withney U Test, * χ^2^ Test)
Age/years	73 (IQR 68–81)	77 (IQR 71–79)	75 (IQR 70–79)	0.6455
Female gender	2 (15.38%)	6 (40.00%)	8 (28.57%)	0.3084 *
Length of hospitalization/days	17 (IQR 7–22)	9 (IQR 6–14)	9 (IQR 6–21)	0.2150
History of diabetes	6 (46.15%)	12 (80.00%)	18 (64.29%)	0.1419 *
History of hypertension	7 (53.85%)	11 (73.33%)	18 (64.29%)	0.4978 *
Coronary artery disease	10 (76.92%)	8 (53.33%)	18 (64.29%)	0.3661 *
Congestive heart failure	11 (84.62%)	8 (53.33%)	19 (67.86%)	0.1732 *
Chronic renal disease	11 (84.62%)	8 (53.33%)	19 (67.86%)	0.1732 *
Liver disease	1 (7.69%)	0 (0.00%)	1 (3.57%)	0.3415 *
Systolic blood pressure at admission/mmHg	110 (IQR 100–130)	140 (IQR 120–160)	120 (IQR 110–145)	0.0767
Pulse at admission/ min^−1^	90 (IQR 76–105)	89 (IQR 80–107)	90 (IQR 79–105)	0.7114
Hemoglobin at admission	123 (IQR 99–129)	136(IQR 101–143)	120 (IQR 99–136)	0.4354
Blood glucose at admission/mmol/L	9.0 (IQR 6.6–12.2)	12.2 (IQR 8.0–14.4)	11.5 (IQR 7.2–13.1)	0.0969
Serum creatinine at admission/μg/L	105 (IQR 95–186)	133 (IQR 100–226)	133 (IQR 100–213)	0.4902
APACHE IV score	63 (IQR 56–114)	54 (IQR 38–72)	62 (IQR 49–83)	0.2301
Ejection fraction/%	35 (IQR 25–40)	50 (IQR 35–55)	40 (IQR 35–50)	0.0164
Hypoglycemia in standard glucose monitoring	5 (38.46%)	1 (6.67%)	6 (23.33%)	0.1134 *
Hypoglycemia reading in FGM	12 (92.31%)	6 (40.00%)	18 (64.29%)	0.0129 *
Mean FGM glucose/mmol/L	5.9 (IQR 5.1–8.3)	7.5 (IQR 6.6–9.5)	7.2 (IQR 5.4–9.2)	0.1074
Glucovariability	23.7 (IQR 17.7–29.3)	25.1 (IQR 18.4–29.7)	23.7 (IQR 18.5–29.3)	0.8887
TIR/%	81 (IQR 27–97)	78 (IQR 55–94)	78 (IQR 51–94)	0.8026
TITR/%	68 (IQR 23–79)	57 (IQR 14–68)	65 (IQR 20–79)	0.3524
TBR/%	6 (IQR 3–13)	0 (IQR 0–3)	2 (IQR 0–11)	0.0093
In-hospital insulin use	7(53.85%)	11(73.33)	18 (64.29%)	0.2832 *

Legend: APACHE IV: Acute Physiology and Chronic Health Evaluation (APACHE) IV; FGM: flash glucose monitoring; TIR: Time in range; TBR: Time below range; TITR: Time in tight range; * χ^2^ Test.

**Table 3:** Effects of left ventricular ejection fraction and hypoglycemia in flash glucose monitoring readings on shock occurrence.

Variable	*p*-Value	Odds Ratio	95% Confidence Interval
LVEF	0.0390	0.9058	0.8246–0.9950
Hypoglycemia in FGM	0.0217	20.4481	1.5543–269.0104

Legend: LVEF: left ventricular ejection fraction; FGM: flash glucose monitoring.

**Table 4:** Effects of gender, left ventricular ejection fraction, and hypoglycemia in flash glucose monitoring readings on shock occurrence.

Variable	*p*-Value	Odds Ratio	95% Confidence Interval
LVEF	0.0382	0.8318	0.6988–0.9900
Hypoglycemia in FGM	0.0174	253.7483	2.6420–24370.7646
Gender	0.1323	0.0378	0.0005–2.6912

Legend: LVEF: left ventricular ejection fraction; FGM: flash glucose monitoring.

## Data Availability

The original contributions presented in the study are included in the article, further inquiries can be directed to the corresponding authors.
